# Charge Shielding of PIP_2_ by Cations Regulates Enzyme Activity of Phospholipase C

**DOI:** 10.1371/journal.pone.0144432

**Published:** 2015-12-11

**Authors:** Jong Bae Seo, Seung-Ryoung Jung, Weigang Huang, Qisheng Zhang, Duk-Su Koh

**Affiliations:** 1 Department of Physiology and Biophysics, University of Washington, Seattle, Washington, 98195, United States of America; 2 Division of Chemical Biology and Medicinal Chemistry, University of North Carolina, 120 Mason Farm Rd., Chapel Hill, NC 27599, United States of America; Indiana University School of Medicine, UNITED STATES

## Abstract

Hydrolysis of phosphatidylinositol 4,5-bisphosphate (PIP_2_) of the plasma membrane by phospholipase C (PLC) generates two critical second messengers, inositol-1,4,5-trisphosphate and diacylglycerol. For the enzymatic reaction, PIP_2_ binds to positively charged amino acids in the pleckstrin homology domain of PLC. Here we tested the hypothesis that positively charged divalent and multivalent cations accumulate around the negatively charged PIP_2_, a process called electrostatic charge shielding, and therefore inhibit electrostatic PIP_2_-PLC interaction. This charge shielding of PIP_2_ was measured quantitatively with an *in vitro* enzyme assay using WH-15, a PIP_2_ analog, and various recombinant PLC proteins (β1, γ1, and δ1). Reduction of PLC activity by divalent cations, polyamines, and neomycin was well described by a theoretical model considering accumulation of cations around PIP_2_ via their electrostatic interaction and chemical binding. Finally, the charge shielding of PIP_2_ was also observed in live cells. Perfusion of the cations into cells *via* patch clamp pipette reduced PIP_2_ hydrolysis by PLC as triggered by M_1_ muscarinic receptors with a potency order of Mg^2+^ < spermine^4+^ < neomycin^6+^. Accumulation of divalent cations into cells through divalent-permeable TRPM7 channel had the same effect. Altogether our results suggest that Mg^2+^ and polyamines modulate the activity of PLCs by controlling the amount of free PIP_2_ available for the enzymes and that highly charged biomolecules can be inactivated by counterions electrostatically.

## Introduction

Electrostatic interaction is one of the most important forces to mediate intramolecular and intermolecular interactions for determining the structure, dynamics, and function of biomolecules [1]. In an aqueous solution, ions and small molecules of opposite charges accumulate around a highly charged biomolecule, resulting in electrical neutralization of the biomolecule [2]. This 'electrostatic charge shielding' can modulate the activity of biomolecules and their availability to partners. For example, shielding of negatively charged phosphates in nucleotides by cations such as Mg^2+^ contributes to the formation of DNA structure [3] and folding of RNA [4]. Since the local potential around a charged molecule determines the degree of counterion accumulation, the shielding effect is more pronounced with highly charged molecules. The charge valence of the counterions is equally important, i.e. divalent cations are more efficient than monovalent cations in shielding of negatively charged molecules. For example, phosphatidylinositol 4,5-bisphosphate (PIP_2_) on the inner leaflet of the plasma membrane is highly charged due to several negatively charged phosphate groups and therefore generates a strong local potential. In aqueous solution, the negative potential around PIP_2_ accumulates cations; this counterion layer is called an 'ion cloud' or 'double layer' [2,5]. PIP_2_ electrostatically interacts less with other neighboring PIP_2_ and protein partners beyond the double layer. By this effect, divalent cations were shown to affect PIP_2_ distribution on the plasma membrane [6]. PIP_2_ interacts with and regulates many proteins such as protein kinase C (PKC) and several ion channels and pumps [7,8]. Therefore the charge shielding effect by cations was well demonstrated with PIP_2_-dependent ion channels such as KCNQ potassium and some transient receptor potential (TRP) channels [9,10].

In addition to the electrostatic accumulation, counterions can be concentrated around charged molecules due to specific binding between them. This interaction is chemical in nature and therefore different divalents of the same charge can interact with negatively charged ions with different binding affinities. It is important to mention that, regardless of the mechanism for accumulation, the counterions neutralize a molecule and interfere with its interaction with other partners electrostatically.

Phospholipase C (PLC) hydrolyzes PIP_2_ to generate inositol 1,4,5-triphosphate (IP_3_) and diacylglycerol (DAG), which subsequently increase cytosolic Ca^2+^ concentration and activate PKC, respectively [8]. Ca^2+^ then regulates a plethora of cell functions such as vesicle trafficking, ion channel conductance, proliferation, differentiation, neurotransmission, and endocrine function [11,12]. Up to now, 13 mammalian PLC isozymes have been identified and divided into six classes: PLCβ1–4, PLCγ1–2, PLCδ1, 3, 4, PLCε, PLCζ, and PLCη1–2 [11,12]. For the catalytic function of PLC, the enzymes have to be located at the plasma membrane where their substrate PIP_2_ is present. This membrane docking of PLC is mediated mostly by their pleckstrin homology (PH)-domain, which has homology sequences of approximately 130 residues in most eukaryotic PLCs [11,13]. Interestingly, nuclear magnetic resonance (NMR) and X-ray crystallography studies revealed that PH-domain structures from 13 different proteins have an identical core β-sandwich fold and more structural than sequence homology [13]. The basic amino acids in the three variable loops between the β sheets of PH domain mediate the electrostatic interaction with PIP_2_ through the formation of a positively charged surface of its three variable loops [13]. Modulating effects of divalent cations or polyamines on PLC enzymes activity were reported in several studies without the molecular mechanism being understood [14–20].

Here, we tested whether electrostatic charge shielding of PIP_2_ by cations regulates the activity of PLCs in *in vitro* conditions using different subtypes of recombinant PLC proteins and analyzed our experimental results with a mathematical model in a more quantitative way. We also examined whether the charge shielding effect of PIP_2_ occurs in intact cells.

## Materials and Methods

### Measurement of PLC Enzyme Activity

To estimate PLC activity we used a fluorogenic reporter substrate, WH-15 [21–23]. Its use for PLC was validated by comparing the results from the traditional PIP_2_ assay, using PIP_2_-containing liposome and radiolabeling of the head group of PIP_2_ ([^3^H] PIP_2_) [21–23]. WH-15 (KXTbio, Durham, NC) was dissolved in an assay solution containing 50 mM HEPES, 70 mM KCl, 3 mM CaCl_2_, 3 mM EGTA, 2 mM DTT, and 0.04 mg/mL fatty acid-free BSA (pH 7.3 adjusted with KOH, 16.4 μM free [Ca^2+^]). The ionic strength of the solution was 0.073. For different free Ca^2+^ concentrations in the assay solutions, CaCl_2_ and EGTA were mixed based on the calculation with the Maxchelator program (http://maxchelator.stanford.edu). The change of free [Ca^2+^] in PLC assay solution was marginal with the addition of MgCl_2_ and increased proportionally with the addition of CaCl_2_. Free concentrations of these two divalent cations were almost equal to the amounts added. However, when BaCl_2_ was added to the assay solution, the free [Ca^2+^] was increased, due to the significant binding of Ba^2+^ ions to EGTA. The binding reduced free [Ba^2+^] by less than 10% (S1 Table for details). Recombinant human PLCβ1 and PLCδ1 proteins were purchased from OriGene (Rockville, MD) and human PLCγ1 was kindly provided by from Dr. John Sondek (University of North Carolina). Because the enzyme becomes unstable during the process, we took care to minimize freeze-thaw cycles. To estimate the charge shielding of PIP_2_ we first preincubated PLC enzymes with divalent cations, polyamines, or neomycin for 15 min and then added 30 μM WH-15 to start the enzyme reaction. The amounts of PLC proteins are: PLCβ1 20 ng/rxn; PLCγ1 10 ng/rxn; PLCδ1 30 ng/rxn. The fluorescence intensity of 6-aminoquinoline was measured after further incubation for 90 and 60 min for PLCβ1 and PLCδ1 at room temperature, respectively. Incubation time was 12 min for PLCγ1 because of its high enzyme activity (Figure B in S1 Fig). The WH-15 assay was performed in 20 μL total volume in Corning 384-well microplates. The fluorescence intensity was measured using BioTek Synergy-4 microplate reader with excitation at 355 nm and emission at 535 nm. The rate of WH-15 hydrolysis by PLC was calculated using a standard curve for the fluorescence intensity of 6-aminoquinolin (Sigma-Aldrich, St. Louis, MO).

### Mathematical Model

To describe the hydrolysis of PIP_2_ by PLC, we first considered free PIP_2_ concentration ([Free PIP_2_]) accessible by PLC. [Free PIP_2_] is reduced by the presence of neighboring cations. These cations are accumulated around PIP_2_ and reduce the electrostatic interaction between PIP_2_ and its partner proteins such as PLC ('electrostatic charge shielding'). The degree of cation accumulation is determined by the electric potential from PIP_2_ and charge valence of the cations, as described by a previous mathematical model [1]. In contrast to the Gouy-Chapman model, a PIP_2_ analog, WH-15, was not incorporated into lipid layer in our in vitro assay system. Rather, the PIP_2_ analog in our model is regarded as a point charge with a specific negative potential. Therefore, the effective cation concentration ([Cation]_eff_) existing around PIP_2_ was described with the Boltzmann equation as below.

$${\left[\text{Cation}\right]}_{\text{eff}}=\left[\text{Cation}\right]\;\times \text{ exp}\left(-\left(\text{z}*\text{q}*\Psi \right)/\text{kbT}\right)$$ (1)

where [Cation] is the bulk concentration, z is the valence of cations, q is the electric charge of one monovalent ion, Ψ is the local potential generated by PIP_2_, k_B_ is the Boltzmann constant, and T is the absolute temperature. Since the accumulation of cations increases exponentially as z increases, we have considered the effect of divalent and polyvalent ions only but not monovalent.

This equation was derived for small cations such as monovalent and divalent ions with all charges concentrated in a small spatial volume. For the large polyamines and neomycin, we considered the size effect to estimate the effective polyamine concentration accumulated around PIP_2_ to explain their dose-response curves.

$${\left[\text{Cation}\right]}_{\text{eff}}=\left[\text{Cation}\right]\;\times \text{ exp}\left(-\left(\left(\text{z}*\text{Y}\right)*\text{q}*\Psi \right)/\text{kbT}\right)$$ (2)

where Y is the factor to describe limited accumulation of the large organic ions around PIP_2_ (crowding effect) and charge distribution over polycationic ions (charge separation effect). The Y factor was determined as 0.12 by comparing putrescine^2+^ and Mg^2+^ effects on PLCβ1. Putrescine has the same charge valence (+2) but it is much larger than Mg^2+^ ion in size. We kept the same Y factor for other polyamines and neomycin for simplicity. The value for **Ψ** at our experimental condition is not well defined. Recently it has been estimated to be -25 mV with a molecular dynamics simulation [24], agreeing with the previous experimental measurements [25]. In fact, the local potential of PIP_2_ is a function of the distance from PIP_2_ and dependent on the orientation of PIP_2_ as well. In our simulation without considering the spatial aspect of cation accumulation, we adapted the suggested value, **Ψ** = -25 mV.

Next, we calculated free PIP_2_ using a Hill-type equation to describe 0 and 100% charge shielding observed at zero and very high concentration of cations, respectively.

$$\left[{\text{Free PIP}}_{2}\right]={\left[{\text{PIP}}_{2}\right]}_{\text{total}}\;\times \;\left(\frac{\text{L}}{\text{L}+{\left[\text{Cation}\right]}_{\text{eff}}}\right)$$ (3)

where [PIP_2_]_total_ is the total PIP_2_ concentration, and L is the half-maximal cation concentration for the charge shielding. As an example, we estimated L value with Mg^2+^ ion considering its effect on three PLC enzymes. The averaged L was 32 mM and the value for polyvalents was reduced proportionally to their valence. As expected, L is determined by the local potential of PIP_2_ critically, i.e. 2.7 times increase of L value with two-fold increase of local PIP_2_ potential.

We also had to consider binding between PIP_2_ and cations as indicated by the differential effect by divalent ions. Therefore [Free PIP_2_] is determined by the cation concentration accumulated around and bound to PIP_2_ molecule.

$$\left[{\text{Free PIP}}_{2}\right]={\left[{\text{PIP}}_{2}\right]}_{\text{total }}\times \;\left(\frac{\text{L}}{\text{L}+{\left[\text{Cation}\right]}_{\text{eff}}}\right)\times \;\left(\frac{1}{1+\text{K}*{\left[\text{Cation}\right]}_{\text{eff}}}\right)$$ (4)

where K, the association constant for binding effect [26], was 4.6 x10^2^ M^-1^ (Ba^2+^), 1.9 x10^2^ M^-1^ (Ca^2+^) and 1 M^-1^ (Mg^2+^) at fixed L value which was estimated with Mg^2+^ in Eq 3.

For the incomplete inhibition of PLC by high concentrations of neomycin, we used W factor.

$$\left[{\text{Free PIP}}_{2}\right]=\left({\left[{\text{PIP}}_{2}\right]}_{\text{total}}-\text{W}\right)\;\times \;\left(\frac{\text{L}}{\text{L}+{\left[\text{Cation}\right]}_{\text{eff}}}\right)\times \;\left(\frac{1}{1+\text{K}*{\left[\text{Cation}\right]}_{\text{eff}}}\right)+W$$ (5)

We do not know yet the molecular mechanism of the steady-state activity of all PLC subtypes observed only with neomycin. Possibly the molecule is large, even larger than the head group of PIP_2_ and therefore there may be steric hindrance between neomycin molecules. Alternatively the accumulation of neomycin around PIP_2_ is limited due to self-repulsion of the neomycin because it has a high charge density. Whichever mechanism applies, the leaking electric field from PIP_2_ will be recognized by PLC, and thereby the enzyme catalyzes the PIP_2_, even at the high concentration of neomycin. The fitting of the neomycin data yielded a W factor of 4.1.

Finally, hydrolysis of PIP_2_ by PLC enzyme is mediated by two simple linear reactions including binding of PIP_2_ to PLC and then catalytic hydrolysis of PIP_2_ by PLC. Therefore a normalized PLC enzymatic activity is described by,

$$\text{Normalized PLC enzyme activity}=\left(\frac{\left[{\text{Free PIP}}_{2}\right]}{\left[{\text{Free PIP}}_{2}\right]+{\text{K}}_{\text{d}}}\right)\times \text{ N }\times \;k\text{ca}$$ (6)

where N is a normalization constant because enzyme activity measured experimentally was normalized to the maximum activity. We assumed that the catalytic activity of PLC is not affected by divalent ions, i.e. the microscopic catalytic reaction rate constant (*k*_cat_) remains the same. The effective dissociation constant between PIP_2_ and enzyme (K_d_) was fixed for all enzymes at 50 μM. Based on the previous study [22], if *k*_cat_ << *k*_dissociation_ (dissociation rate constant of PIP_2_ to enzyme), we can assume K_d_ *≈* K_m_. Michaelis-Menten constants (K_m_) were measured as 86, 30, and 50 μM for PLCβ2, PLCδ1, and PLCγ1, respectively [22]. Interestingly, when we use a K_m_ value same as K_d_, we obtained similar L value (~30 mM) compared to the case with fixed K_d_ = 50 μM for all enzymes, suggesting that electrostatic accumulation (L) is not strongly affected by the K_d_. All simulations were performed with Igor software (WaveMetrics).

### Cell Culture and Transfection

A HEK293-TRPM7 cell line kindly provided by Dr. Andrew M. Scharenberg (Children's hospital in Seattle) was grown in Dulbecco's modified Eagle's medium (DMEM) supplemented with 10% fetal bovine serum, 0.2% penicillin/streptomycin, blasticidin (5 μg/ml), and zeocin (0.4 mg/ml) [27]. Expression of TRPM7 channel was induced 1 d before use by adding 1 μg/ml tetracycline (Sigma-Aldrich) to the culture medium. HEK293-tsA201 cells (Sigma-Aldrich) were cultured in DMEM supplemented with 10% FBS and 0.2% PS at 37°C and 5% CO_2_. Transfection was performed with Lipofectamine 2000 (Invitrogen) according to the manufacturer’s specifications with 1 μg expression vector DNA of M_1_ muscarinic receptor (M_1_R) and 0.5 μg DNA of eYFP-PH-PLCδ1 (PH-YFP) per 35-mm dish. Cells were plated onto poly-L-lysine-coated glass chips 1 or 2 days before the experiment.

### Manipulation of Cations in the Cytoplasm and Measurement of PIP_2_ Hydrolysis

Cells were patch clamped using an EPC 9 amplifier (HEKA Elektronik, Lambrecht/Pfalz, Germany). Patch electrodes had resistances between 7 and 10 MΩ when filled with internal solution. External normal Ringer’s solution contained (in mM): 137.5 NaCl, 2.5 KCl, 1 MgCl_2_, 2 CaCl_2_, 10 glucose, and 10 HEPES; divalent-free external solution, 147.5 NaCl, 2.5 KCl, 10 glucose, and 10 HEPES; 10 mM MgCl_2_ external solution, 140 NaCl, 2.5 KCl, 10 MgCl_2_, 10 glucose, and 10 HEPES; 10 mM BaCl_2_ external solution, 140 NaCl, 2.5 KCl, 10 BaCl_2_, 10 glucose, and 10 HEPES. All external solutions were adjusted to pH 7.3 with NaOH. The pipette solution contained (in mM): 160 KCl, 10 HEPES, 0.1 BAPTA, 3 ATP, 0.1 GTP, 1 MgCl_2_, adjusted to pH 7.2 with KOH. For the accumulation of divalent ions through TRPM7, external 10 mM MgCl_2_ or BaCl_2_ containing solutions were perfused at a holding potential of -80 mV.

Alternatively, we dialyzed the divalent and polycations using patch pipettes with different internal solutions, and used the external normal Ringer’s as bath solution. Patch electrodes had a resistance between 4 and 6 MΩ when filled with internal solution containing (in mM): 160 KCl, 10 HEPES, 0.1 BAPTA, 3 ATP, 0.1 GTP, 1 MgCl_2_, adjusted to pH 7.2 with KOH. For internal solutions containing 3 or 10 mM MgCl_2_, KCl was reduced to 155 and 145 mM, respectively, to adjust osmolarity. Spermine and neomycin were added to the internal solution containing 1 mM MgCl_2_. To accelerate dialysis of divalent or polycations, we used electrophoresis of the ions driven by potassium currents [28]. The current conducted by endogenous potassium channel was repetitively activated by voltage jumps from -80 mV (500 ms) to + 80 mV (2 s). Under that voltage clamp condition, the same current flows from the patch pipette to the cell and the current is mediated by the flow of positive ions including the cations under investigation.

PIP_2_ hydrolysis by PLCs was monitored using eYFP-PH-PLCδ1 (PH-YFP, in real topology YFP attaches to the N-terminal side of PH domain). PH-YFP was excited at 514 nm and emission was detected at 525–600 nm using a Zeiss 710 laser-scanning confocal microscope. Upon activation of PLC, the probe translocates to the cytoplasm together with IP_3_ as described previously [10]. The intensity of PH-YFP at the specific regions of interest in the cytosol was analyzed using ImageJ software (NIH) after normalization to reduce cell-to-cell variation. To estimate PIP_2_ hydrolysis, we measured the percent difference between basal and peak fluorescences before and after oxotremorine M (Oxo-M) treatment.

All experiments were performed at room temperature.

### RNA isolation, cDNA synthesis, and quantitative real-time PCR (Q-PCR)

Total RNA was isolated from HEK293-tsA201 and stable HEK293-TRPM7 cells with Pure Link ^®^ mini kit (Invitrogen, Grand Island, NY) according to the manufacturer’s instruction. First-strand cDNA was synthesized by reverse transcription of 2 μg of total RNA with SuperScript^®^ III First-Strand Synthesis System (Invitrogen) following standard protocols. Q-PCR was performed on MX3000P^®^ system (Stratagene) with iTaq Universal SYBR^®^ Green Supermix (Bio-Rad) according to the manufacturer's instruction. The reaction was conducted as follows; 95°C for 3 min followed by 40 repetitive thermal cycles (95°C for 15 s, 55°C for 30 s, 72°C for 20 s). Primers were purchased from Integrated DNA Technologies. Primer sequences were as follows [29]: PLCβ1 (sense 5’- AGC TCT CAG AAC AAG CCT CCA ACA-3’ antisense 5’-ATC ATC GTC GTC GTC ACT TTC CGT-3’); PLCβ2 (sense 5’-AAG GTG AAG GCC TAT CTG AGC CAA-3’ antisense 5’-CTT GGC AAA CTT CCC AAA GCG AGT-3’); PLCβ3 (sense 5’-TAT CTT CTT GGA CCT GCT GAC CGT-3’ antisense 5’-TGT GCC CTC ATC TGT AGT TGG CTT-3’); PLCβ4 (sense 5’-GCA CAG CAC ACA AAG GAA TGG TCA-3’ antisense 5’-CGC ATT TCC TTG CTT TCC CTG TCA-3’); GAPDH (sense 5’-CGA GAT CCC TCC AAA ATC AA-3’ antisense 5’-GTC TTC TGG GTG GCA GTG AT-3’). The message level of each gene was normalized to that of GAPDH.

### Statistical analysis

All numerical values in the text and figures are given as mean ± SEM. *n* and *N* denote the numbers of analyzed samples and cells, respectively. Statistical significance was determined by Student’s *t*-test, and *P* < 0.05 was considered significant.

## Results

### Divalent and Multivalent Cations Reduces PLCβ1 Activity

**Fig 1:**
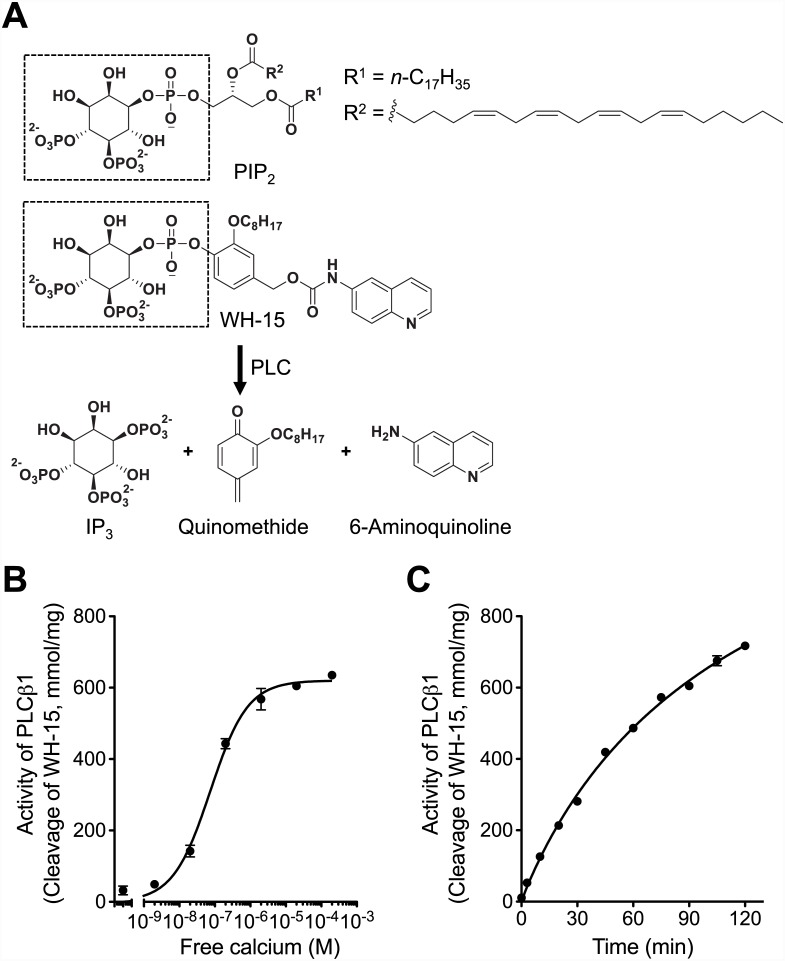
*In vitro* assay of PLC enzyme activity. (A) WH-15, a PIP_2_ analog, is cleaved by PLCs into inositol 1,4,5-triphosphate (IP_3_), a quinomethide derivative, and 6-aminoquinoline. Fluorescence from 6-aminoquinoline is used to estimate PLC activity. WH-15 has the same inositol as PIP_2_ that is recognized and cleaved by PLC (dotted rectangle box). (B) Ca^2+^-dependent activation of PLCβ1. WH-15 (30 μM) was hydrolyzed by recombinant PLCβ1 protein (20 ng) at different free Ca^2+^ concentrations for 90 min. (C) Real-time PLCβ1 activity was measured with a fixed free Ca^2+^ concentration at 16.4 μM. Error bars for many points are smaller than the symbol size. *n* = 3 for each condition.

To address our hypothesis that cations screen PIP_2_ electrostatically, we carried out *in vitro* measurements of several recombinant PLC proteins using a recently developed reporter. WH-15 is a water-soluble and fluorogenic analog of PIP_2_ designed to report the catalytic activity of PLCs and revealed similar kinetic properties (*K*_m_ and *V*_max_ values) to those determined with PIP_2_ solubilized in mixed micelles [22]. Hydrolysis of this reporter by PLCs also was dependent on calcium concentration as that of endogenous PIP_2_ (Fig 1 and S1 Fig) [21–23]. The reporter can be cleaved by PLCs into IP_3_, a quinomethide derivative, and fluorescent 6-aminoquinoline (Fig 1A). Therefore PLC activity can be precisely estimated by measuring the fluorescence from 6-aminoquinoline.

First, as a control experiment, we tested Ca^2+^-dependence of PLCβ1 at different free Ca^2+^ concentrations ([Ca^2+^], 2 nM ~ 200 μM) (Fig 1B). Activity of PLCs was calculated by determining the conversion rate of WH-15 molecules using a standard curve of fluorescence intensity of 6-aminoquinoline. As shown in Fig 1B, Ca^2+^ stimulated PLCβ1 to cleave WH-15 with saturation at 20 μM [Ca^2+^] consistent with PIP_2_ hydrolysis by PLC in the previous studies [19,20]. At this assay condition, PLCβ1 cleaved its substrate slowly (Fig 1C) at a rate similar to PLCδ1 (Figure A in S1 Fig). In contrast, PLCγ1 exhibited a higher activity and hydrolyzed all supplied WH-15 molecules within around 30 min (Figure A in S1 Fig). The activity of PLCβ1, PLCγ1, and PLCδ1 was 9.4 ± 0.1, 148.9 ± 2.9, and 6.2 ± 0.4 mmol/min/mg, respectively (Figure B in S1 Fig). The result indicates a high precision of our *in vitro* PLC assay and an easy control of the components included in the reaction.

**Fig 2:**
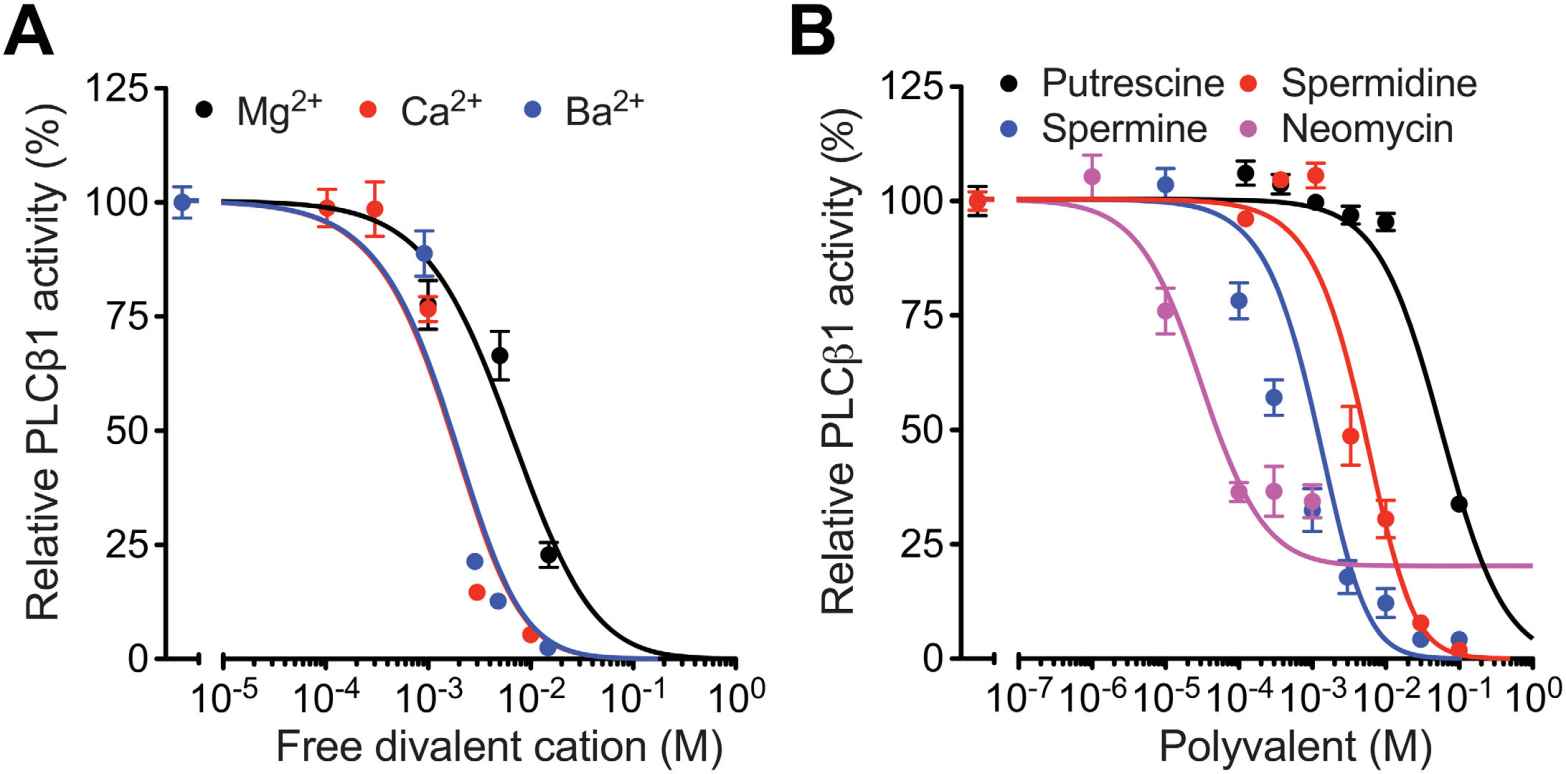
Enzyme activity of PLCβ1 in the presence of multivalent cations. Activity of PLCβ1 was measured and calculated as described in Materials and Methods in the presence of different concentrations of divalent cations (A) and polyamines or neomycin (B). Free Ca^2+^ concentration was set as 16.4 μM except the measurements using different CaCl_2_ concentrations. Symbols and lines are experimental data and fitting with our mathematical model, respectively. *n* = 4–8 for each condition.

Next we measured activity of PLCβ1 in the presence of different concentrations of divalent and polyvalent cations. All divalent cations reduced the activity of PLCβ1 enzymes dose-dependently (Fig 2A). For this result, we performed a control experiment. The inhibition was not due to the decrease of free [Ca^2+^] and the consequent drop in Ca^2+^-dependent activity of PLCβ1, because free [Ca^2+^] increased in all test solutions (S1 Table). For example, when 15 mM MgCl_2_ or BaCl_2_ was added to the assay solution, the free [Ca^2+^] was 24.2 or 333.2 μM, respectively, based on our calculation with the Maxchelator program (http://maxchelator.stanford.edu). This is the concentration range at which the inhibition does not occur yet (Fig 2A).

In addition, the inhibition of PLCβ1 was variable with different cations (Ba^2+^ = Ca^2+^ > Mg^2+^) (Fig 2A). Again this differential effect could not be explained by different free [Ca^2+^] in the test solutions, because it was greater than the saturating level but less than the critical concentration for the charge screening for all measurements. Therefore, we interpret that this observation is presumably due to the different accumulation of divalent cations around PIP_2_ with their different chemical binding affinities (see Discussion for more details).

Since electrostatic charge shielding of negatively charged ions by cations is critically dependent on charge valence of the cations (Eq 1), we tested diverse polyamines and neomycin. As expected, cations with higher charges had a stronger effect on PLCβ1: neomycin (+6) > spermine (+4) > spermidine (+3) > putrescine (+2) (Fig 2B), in line with the idea that the cations accumulate around and screen PIP_2_ electrostatically. In addition, we observed two interesting phenomena; 1) neomycin did not inhibit the enzyme activity completely and 2) even with the same valence, putrescine was less efficient than divalent cations in reducing PLCβ1 activity. These results, together with divalent-dependent effectiveness (Fig 2A), are not predicted by the simple charge shielding effect, suggesting that other factors are involved as discussed below. Nevertheless reduction of PLCβ1 activity by divalent and multivalent cations in a charge-dependent way agrees to our hypothesis that the cations shield PIP_2_ electrostatically. If the charge shielding of PIP_2_, the substrate of PLCβ1, is a critical determinant for the reduction of enzyme activity, we expect the same effect with other subtypes of PLC.

### Divalent and Multivalent Cations Reduce the Activity of PLCγ1 and PLCδ1

**Fig 3:**
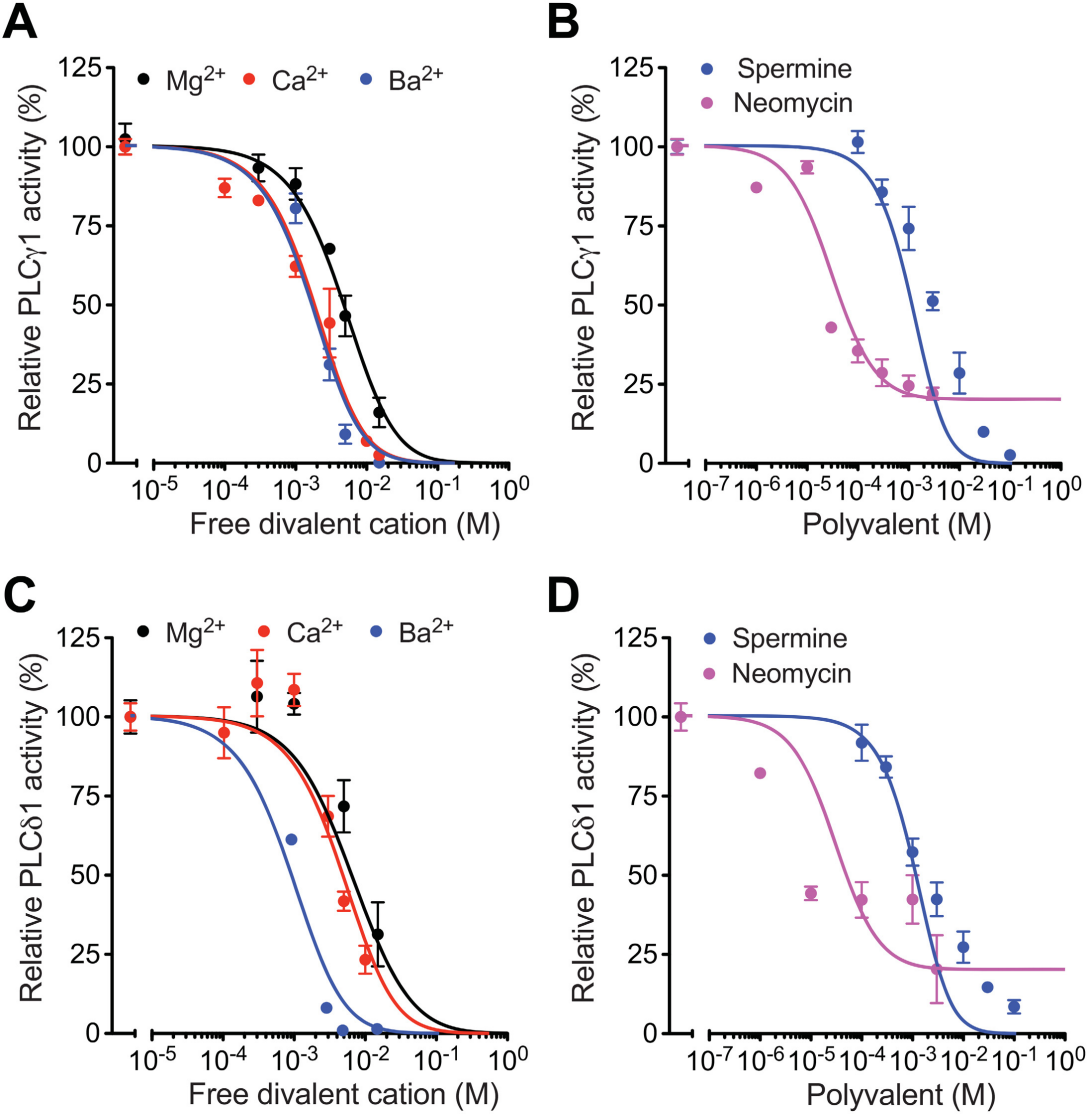
Enzyme activity of PLCγ1 and PLCδ1 in the presence of multivalent cations. Activity of PLCγ1 (A, B) and PLCδ1 (C, D) was measured as PLCβ1 in Fig 2. Symbols and lines are experimental data and fitting with the mathematical model, respectively. *n* = 4–8 for PLCγ1 and *n* = 2–8 for PLCδ1.

PLCγ1 and PLCδ1 also hydrolyze PIP_2_ [11,12]. Assay of enzyme activity with recombinant PLCγ1 and PLCδ1 shown in Fig 3 indicated similar results as PLCβ1 with some difference in detail: PLCγ1 (IC_50_ = 4.0 mM) and PLCδ1 (IC_50_ = 1.7 mM) were slightly less sensitive to spermine (Fig 3A and 3B) compared to PLCβ1 (IC_50_ = 0.4 mM). In addition, PLCδ1 (IC_50_ = 6 μM) was more sensitive to neomycin compared to other PLCs (IC_50_ = 11 or 24 μM for PLCβ1 or PLCγ1, respectively; Fig 3C and 3D). Interestingly, the inhibitory effect of neomycin saturated beyond certain concentrations for both PLCγ1 and PLCδ1 as found for PLCβ1.

### Mathematical Modeling of PLC Activity in the Presence of Different Cations

To describe the hydrolysis of PIP_2_ by PLC quantitatively, we fitted the *in vitro* data using an empirical model based on modified electrostatic charge shielding (see Methods for details, [26]). For example, the inhibition of PLCβ1 by Mg^2+^ is apparent at [Mg^2+^] > 10 mM (Fig 2A). Our model predicted that Mg^2+^ accumulates around PIP_2_ by electrostatic interaction at a half maximal concentration (L) of 32 mM. For different divalents, we had to consider additional bindings of the cations to PIP_2_, since their accumulation by the electrostatic interaction to PIP_2_ is equal. From the model the effective dissociation constants (1/K) for the binding are 2.1, 5.2, and 1,000 mM for Ba^2+^, Ca^2+^, and Mg^2+^, respectively (Fig 2A). The result suggests that accumulation of Mg^2+^ around PIP_2_ is mainly mediated by electrostatic screening and other divalents accumulate with both electrostatic and chemical interactions. The sequence of the ions to bind to PIP_2_ remains similar for all PLC subtypes, consistent with our hypothesis that the effect of the cations is on PIP_2_, not PLC (Fig 3A and 3C). Interestingly, putrescine with two positive charges was less effective for inhibition of PLC enzyme compared with the Mg^2+^ effect. We reasoned that accumulation of putrescine is less efficient due to larger size than divalent cations (crowding effect) and the effective charge of putrescine, due to the charge distribution in the putrescine molecules (charge separation effect), is less than divalent ions. A smaller Y factor, which reflects crowding and charge separation effects (0.12 compared to Mg^2+^ with 1, see Methods for details) could describe the dose-response curves for PLCβ1 subtype we tested (Fig 2B). Inhibition of PLCβ1 by other polyamines and neomycin was well described by assuming their accumulation around PIP_2_ as determined by their electrostatic charge screening, binding, size effect and repulsion effect. For the binding effect, the effective dissociation constant (1/K in the model) of putrescine, spermidine, spermine, and neomycin with PLCβ1 were 3,600, 19, 10, and 1 mM, respectively. Inhibition of PLCγ1 and PLCδ1 by divalent cations (Fig 3A and 3C), spermine, and neomycin (Fig 3B and 3D) was well described by the same parameters except with different binding affinities of the enzymes to PIP_2_. We found it quite challenging to fit the effect of multiple ions on three subtypes of PLC with the same parameters. The successful fitting of our experimental data with our model, based on charge shielding of PIP_2_, supports our original hypothesis that accumulation of cations around PIP_2_ limits its availability to PLC.

### Loading of Divalent Cations Through TRPM7 Channels Reduces PLC-mediated PIP_2_ Hydrolysis in Cells

To test whether the charge shielding of PIP_2_ can be also observed in live cells, we next performed *in cell* experiments. Transient receptor potential M7 (TRPM7) channels are essential for Mg^2+^ homeostasis in mammals [30] and highly permeable to divalent cations such as Mg^2+^, Ba^2+^, Ni^2+^, Zn^2+^, and Ca^2+^ [31]. Therefore, we used these channels to accumulate divalent cations in HEK293 cells.

**Fig 4:**
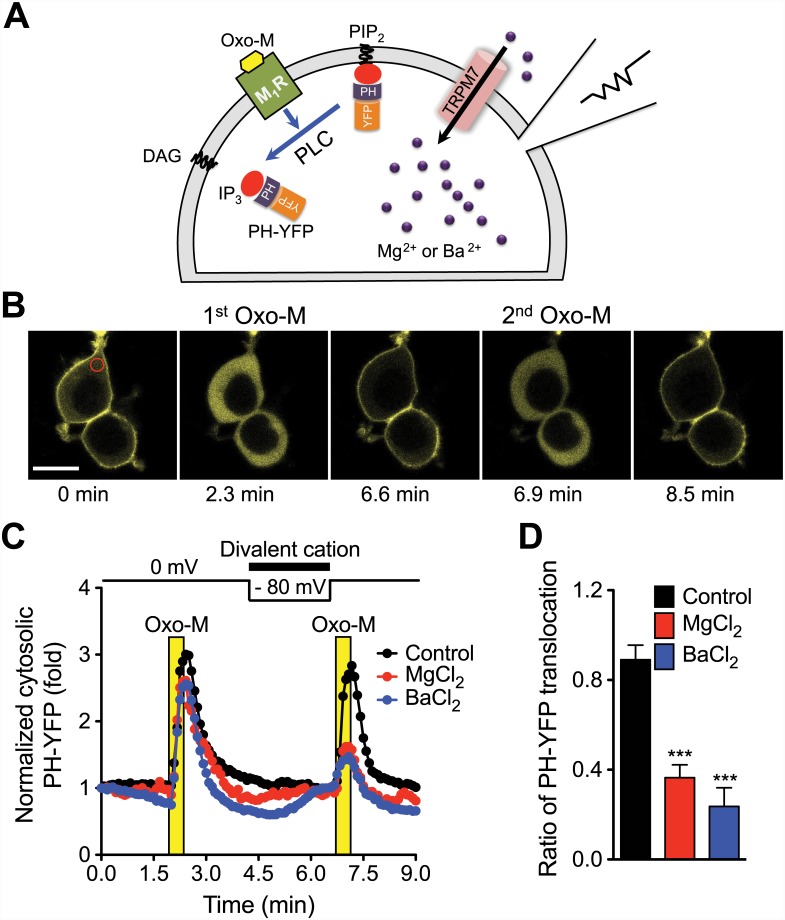
Inhibition of PIP_2_ hydrolysis by Mg^2+^ or Ba^2+^ accumulated into cells through TRPM7. (A) Schematic diagram. HEK293-TRPM7 cells were transfected with M_1_R and PH-YFP and TRPM7 channels were activated by voltage clamp to accumulate divalent. (B) Confocal images of PH-YFP where the fluorescence is coded as yellow. Images are taken before and during application of 1 μM oxotremorine-M (Oxo-M) in control group. Top cell was patched. Region of interest (ROI) used for the PH-YFP translocation analysis is indicated in red circle. Black scale bar indicates 20 μm. (C) The average rate of PIP_2_ hydrolysis by PLC was estimated by the monitoring of translocation of PH-YFP into the cytosol upon activation of M_1_R with 1 μM Oxo-M. For the accumulation of divalent cations into the cells, external solutions containing 10 mM MgCl_2_ (*N* = 5) or 10 mM BaCl_2_ (*N* = 7) were perfused at the indicated time, and their influx through TRPM7 was triggered by a negative membrane potential (-80 mV). Error bars were omitted for clarity. (D) Comparison of PLC activity before and after the divalent accumulation. Percent increase of cytosolic PH-YFP upon 2nd Oxo-M treatment was divided by that of 1st Oxo-M (see Methods for details). The results are mean ± SEM and representative of two independent experiments. *** *P* < 0.001 compared to control group (*N* = 5).

Expression of TRPM7 channel in the HEK293-TRPM7 cell line was induced by tetracycline as confirmed by measuring TRPM7 currents using patch-clamp experiment (S2 Fig). To monitor PIP_2_ hydrolysis by endogenous PLC, we transfected the cells with PH-YFP, a PIP_2_-binding fluorescent probe [10]. Under a confocal microscope, the PH-YFP probe was localized at the PIP_2_-enriched plasma membrane (Fig 4B). When muscarinic receptor 1 (M_1_R) was activated by submaximal 1 μM Oxo-M, PLC cleaved PIP_2_, inducing translocation of the PH-YFP probe to the cytosol. Upon washout of the agonist, PIP_2_ was regenerated and the probe returned to the plasma membrane (Fig 4B and 4C). To test the effects of divalent cations on PLC activity, we loaded them into a cell through TRPM7 channels using a negative membrane potential (-80 mV inside the cell) for 2 min before the second Oxo-M treatment. In control cells perfused with the control external solution without divalent ions, PLC activity monitored by PH-YFP translocation to the cytosol was comparable to that induced by the first Oxo-M application (Fig 4C, 0.89 ± 0.06 fold compared to the first probe translocation, Fig 4D). In contrast, the cytosolic translocation of PH-YFP triggered by M_1_R activation was considerably reduced in the cells loaded with Mg^2+^ (0.36 ± 0.06) and Ba^2+^ (0.24 ± 0.08). This potency is in line with the permeability of TRPM7 of the cations (Ba^2+^ > Mg^2+^) [31]. It should be noted that the effect of Ba^2+^ on PIP_2_ hydrolysis is overestimated due to a spontaneous PH-YFP translocation occurring before Oxo-M treatment. This effect was small but evident with Ba^2+^. We interpret that some PIP_2_ molecules are screened electrostatically by the accumulated divalent cations and no longer available to bind to PH-YFP, promoting the translocation of the probe to the cytosol prior to Oxo-M treatment. In addition, we repeated the same experiment with 1 mM extracellular MgCl_2_, a physiological Mg^2+^ concentration. We did not observe any inhibitory effect (S3 Fig), suggesting that the accumulated [Mg^2+^]_i_ was not sufficient to achieve the critical PIP_2_ screening for PLC activity.

### Accumulation of Mg^2+^, Spermine, and Neomycin Through Patch Pipette Attenuates PLC-mediated PIP_2_ Hydrolysis in Cells

**Fig 5:**
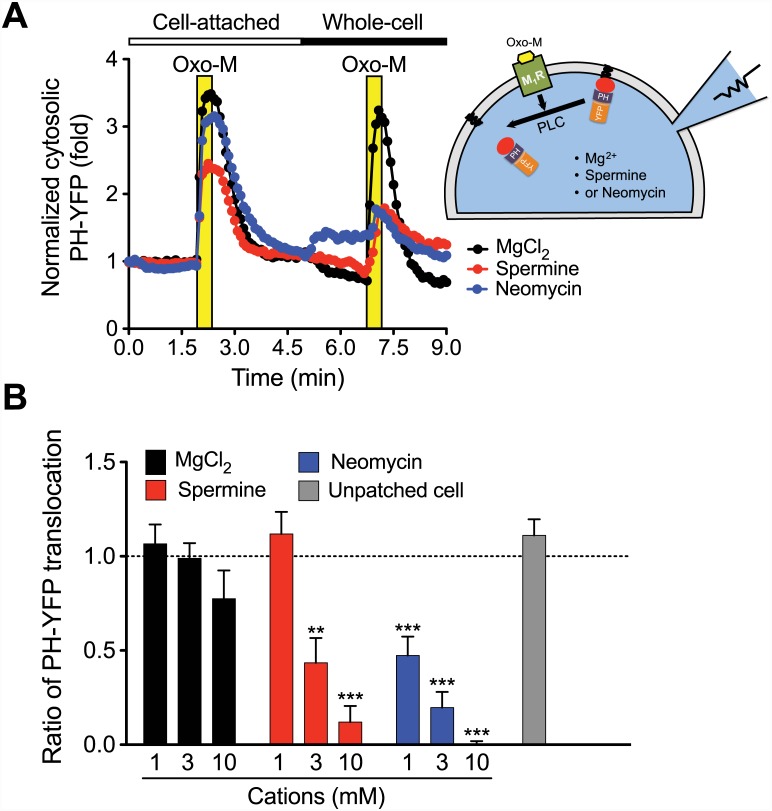
Inhibition of PIP_2_ hydrolysis by Mg^2+^, spermine, or neomycin dialyzed into cells through patch pipette. (A; Right) HEK293-tsA201 cells were transfected with M_1_R and PH-YFP and dialyzed cations through whole-cell patch pipette. (Left) The average rate of PIP_2_ hydrolysis by PLC was estimated as Fig 1. The first Oxo-M response was triggered while being in cell-attached mode. For accumulation of the cations, the membrane patch was ruptured to form the whole-cell configuration and the cations in the pipette solution were dialyzed into the cell. To accelerate the movement of the cations by electrophoresis, the voltage steps to +80 mV from -80 mV for 2 s were repeated. The effects of 3 mM MgC1_2_ (*N* = 6), spermine (*N* = 5), and neomycin (*N* = 4) are shown. Error bars were omitted for clarity. (B) Summary of PLC activity after pipette perfusion of different concentrations of the cations. PIP_2_ hydrolysis in the neighboring unpatched cells was nearly unaltered. The results are mean ± SEM and representative of three independent experiments. *N* = 4–10 for each condition. ** *P* < 0.01 and *** *P* < 0.001 compared to 1 mM MgCl_2_ group (*N* = 10).

Finally, we dialyzed cations with higher valencies such as spermine and neomycin using patch pipettes into HEK293-tsA201 cells. Control PLC activity was measured by Oxo-M treatment and PH-YFP translocation to the cytoplasm in the cell-attached mode (i.e. gigaseal formed). Then the patch membrane was ruptured to form the whole-cell configuration, and divalent Mg^2+^ or multivalent spermine^4+^ or neomycin^6+^, that were included in the patch pipette, were dialyzed into the patched cell. In addition, we applied repetitive voltage jumps to +80 mV to activate endogenous K^+^ current and thereby to promote cellular accumulation of the cations by electrophoresis (see Methods for details). PLC activity, stimulated by the second Oxo-M application after accumulation of the cations, rarely changed compared to the first application when 3 mM Mg^2+^ was included in the patch pipette (Fig 5A, black trace). Unlike the result in Fig 4, perfusion of 10 mM Mg^2+^ through the patch pipette inhibited PIP_2_ hydrolysis slightly but the effect was not statistically significant. This marginal effect is probably due to a lower [Mg^2+^]_i_ reached via simple diffusion through the pipette compared to the Mg^2+^ accumulation through TRPM7 with a strong electromotive potential (i.e. -80 mV). In contrast, the same concentration of spermine or neomycin reduced PH-YFP translocation, suggesting lower PLC activity. In the case of neomycin (Fig 5A), there was PH-YFP translocation, even before the second Oxo-M application as observed with BaCl_2_ (Fig 4C) accumulated through TRPM7 channels. This result may be due to the fact that neomycin^6+^ has higher charge density than that of spermine^4+^ so that only the neomycin-induced PH-YFP translocation is observed before M_1_R activation. In summary, spermine (≥ 3 mM) and neomycin (≥ 1 mM) retarded PIP_2_ hydrolysis significantly in intact cells (Fig 5B). We interpret that the inhibitory effect of polycations is mediated by the screening of PIP_2_. However the same result can be achieved by the inhibition of signaling molecules upstream of PLC. Therefore we measured the interaction between M_1_R and G_αq_ using the fluorescently labeled proteins and fluorescence resonance energy transfer (FRET) analysis. The M_1_R-G_αq_ interaction was not affected by the dialysis of 3 mM spermine through patch pipette (S4 Fig).

**Fig 6:**
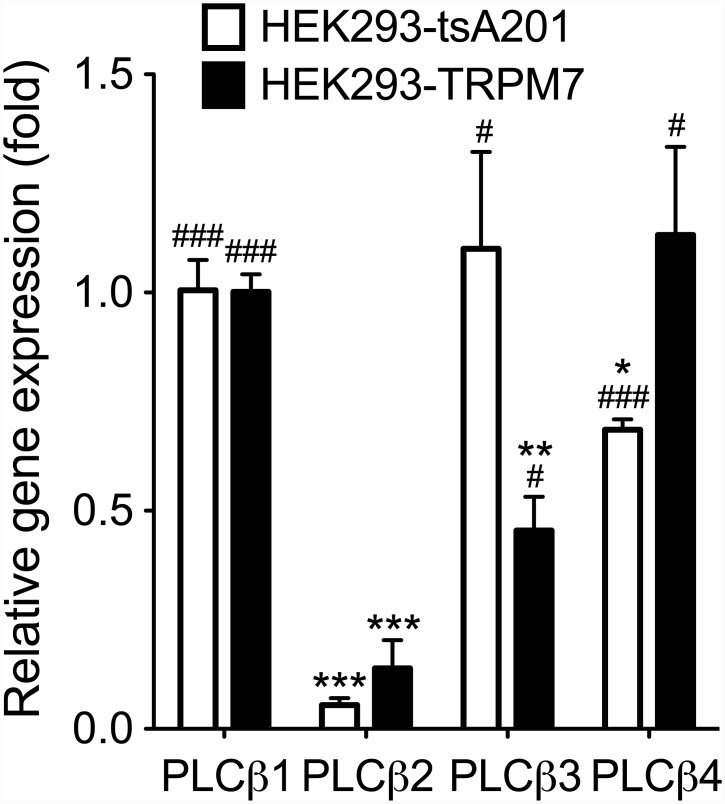
Expression of PLCβ in HEK293-tsA201 and HEK293-TRPM7 cells. Expression level of PLCβ isoforms was analyzed with Q-PCR. Their mRNA levels were normalized to GAPDH and presented as relative to PLCβ1. *n* = 3 for each condition. * *P* < 0.01, ** *P* < 0.01, and *** *P* < 0.001 compared to PLCβ1 group. ^#^ *P* < 0.05 and ^###^ *P* < 0.001 compared to PLCβ2 group.

Since M_1_R is a G_q_-protein coupled receptors (G_q_PCR) and coupled to PLCβ, we speculate the Oxo-M activates this isoform of PLC [32]. Mammalian PLCβ consists of 4 subtypes (PLCβ1–4). We screened the type of PLCβ in our cells using quantitative PCR. PLCβ1 and PLCβ3 were the predominant genes expressed in HEK293-tsA201 cells, whereas PLCβ1 and PLCβ4 were abundant in HEK293-TRPM7 cells (Fig 6). Therefore we speculate that the divalent and multivalent cations inhibited the PIP_2_ hydrolysis mediated by PLCβ1,3,4 enzymes in our *in cell* experiments.

## Discussion

Our quantitative *in vitro* PLC assay and modeling support the hypothesis that divalent and multivalent cations accumulate PIP_2_ and reduce its availability to PLC rather than the direct inhibition of the enzyme by the cations. The evidence is as follows: 1) A variety of PLC enzymes are similarly inhibited by divalent cations, polyamines and neomycin. 2) PIP_2_ probe (PH-YFP) as well is released from the plasma membrane when the di- or multivalent cations are loaded into live cell. 3) Most importantly, the mathematical model based on charge shielding of PIP_2_ and binding affinity of the cations to the PIP_2_, successfully fits dose-response curves of activity of multiple PLC subtypes. Therefore we tentatively conclude that the direct modulation of PLC protein by the cations is marginal and specific for certain cations, if any inhibition is present. In *in cell* experiments, PIP_2_ hydrolysis by PLC was similarly reduced, suggesting that PIP_2_ shielding occurs in more physiological environment as well.

In the following sections, we will review and discuss the evidence for charge shielding of PIP_2_ to reduce the activity of PIP_2_-interacting proteins and the significance of our findings with PLC.

### Electrostatic Charge Shielding of PIP_2_ by Cations

The charge shielding effect by cations depends on the local potential of PIP_2_, bulk concentrations and the valences of the cations, the chemical binding of cations to PIP_2_, and ionic strength of the solution. These factors determine the thickness of the double layer which ranges from a few to several tens of Å. A charged biomolecule beyond the layer does not “feel” the electric field emitted by the shielded biomolecules so that the electrostatic interaction with their partners is compromised. As described above, the valence of the cations is important, because the accumulation of cations around the PIP_2_ increases exponentially with charge numbers so that monovalent ions are far less effective than divalent cations. Therefore we observed the charge shielding of PIP_2_ by divalent cations (Mg^2+^, Ca^2+^, and Ba^2+^) and multivalent cations (> +2).

Previous study demonstrated that polyvalent anionic PIP_2_ can be nonspecifically screened by cations [33]. This charge shielding was well demonstrated with ion channels whose activity needs PIP_2_ [10,33]. For example, PIP_2_-dependent KCNQ1 (KCNE1) and KCNJ1 (Kir2.1) potassium channel activity is inhibited by divalent and trivalent cations [10,34]. In addition, TRP currents including TRPV5 and TRPM7 are reduced by intracellular Mg^2+^ as well as other divalent such as Ba^2+^, Sr^2+^, Mn^2+^, and Zn^2+^ through charge shielding of PIP_2_ by cations in rat basophilic leukemia and T-lymphocyte and in heterologous expression systems [9,27,33]. It was also suggested that divalent ions bind to PIP_2_ in addition to their charge shielding effect [35]. This interaction between PIP_2_ and divalent ions is chemically specific, i.e. different ions have different binding affinities as we observed with *in vitro* PLC assays. Recent studies also suggest that proteins with positively charged amino acids such as myristoylated alanine-rich C kinase substrate (MARCKS), can screen PIP_2_ [36,37]. This type of charge shielding is regarded to be different from that induced by small ions in solutions. For example, the expected length of a peptide of MARCKS (151–175) used in the studies is ~ 10 nm, whereas the ionic radius of Mg^2+^ is 0.1 nm. However the final result is the same: shielding of negative charges in PIP_2_, its neutralization, and preventing PIP_2_ from interacting with its partner molecules.

### PLCs are Regulated by Divalent Cations

Two physiologically important divalent cations, Ca^2+^ and Mg^2+^, play important roles for diverse cell functions such as enzyme activity, cell growth, cell migration, bone formation, hormone secretion, muscle contraction, neural excitability, and blood coagulation [38]. The free intracellular concentrations range from 100 nM to a few μM for Ca^2+^ and from 100 μM to low mM for Mg^2+^ [39,40]. Unlike dynamic modulation of [Ca^2+^]_i_ by diverse extracellular inputs [41], regulation of [Mg^2+^]_i_ is not well studied. However, some studies revealed that [Mg^2+^]_i_ can be regulated in specific cell types expressing TRPM channels [42].

Divalent cations control PIP_2_ clustering on lipid membranes through electrostatic interaction between divalent cations and anionic groups of PIP_2_, i.e. charge shielding and neutralization of PIP_2_ [6,43]. This result suggests that divalent cations can also affect the interactions between PIP_2_ molecules. Soon after the purification and cloning of PLC subtypes, it was realized that PLC activity is stimulated by low concentrations of Ca^2+^ at < 0.1 mM [19,20]. Some studies also showed that higher Ca^2+^ > 0.1 mM inhibit the enzyme activity by an unknown mechanism, resulting in a bell-shaped dose-response curve. For example, purified bovine brain PLCs are activated by Ca^2+^ until 100 μM and inhibited above a few 100 μM Ca^2+^ [19,20]. The phenomena are also empirically observed at high concentrations of Ca^2+^ with recombinant phosphoinositide-specific PLC (PI-PLC) isoforms from plants and bovine brain [15,17]. In spite of repeated observation, the molecular mechanism of the inhibitory effect remained not identified. We now postulate that the limited availability of PIP_2_ by charge shielding is the major cause for the slow down in enzyme activity.

Consistent with these previous reports, our experimental results showed that divalent cations overall inhibit the activity of PLCs in the millimolar range with different potencies. For example, our results suggest that the charge shielding effect of Mg^2+^ is weaker than that of other divalent cations, Ca^2+^ and Ba^2+^. This difference was detected with all PLCs tested, suggesting that divalent ions bind to PIP_2_ with different binding affinities. In other words, chemical binding results in more accumulation of certain divalent ions around PIP_2_ and further reduction of PIP_2_ availability to PLC. This kind of electrostatic interaction of chemical nature exhibits different binding affinities between cations of the same charge as demonstrated with the interaction between alkali metal cations and ion-selective glass [44].

The final profile of cations around PIP_2_ appears to be determined by both the electrostatic interaction and chemical binding.

### PLCs are Regulated by Multivalent Cations

Polyamines including putrescine, spermidine, and spermine are ubiquitous in all living organisms and play crucial roles in proliferation, migration, transformation, and apoptosis [45,46]. Putrescine converted by ornithine decarboxylase from ornithine and then this diamine is sequentially converted into spermidine and spermine by spermidine synthase and spermine synthase, respectively [45,46]. The intracellular concentration of polyamines is often in the millimolar range and tightly regulated. The free concentration and binding to DNA and RNA fluctuate along the cell cycle as being highest at G2 phase [46]. Dysfunction of polyamine metabolism is invoked in cancer, Parkinson’s disease, Alzheimer’s disease, cataract formation, and multiple sclerosis [47]. For example, the level of polyamines, especially spermidine and spermine increase in cancer cells. Nonetheless, the biological and physiological function and mechanisms of polyamines are not fully understood. By reviewing the effect of spermine and spermidine involving PIP_2_, Coburn (2009) suggested that diverse cellular functions affected by polyamines could be mediated by screening of PIP_2_ and consequent reduction of activity of PIP_2_-sensitive proteins [45]. Our study tested this hypothesis with PLC.

Polyamines and neomycin reduced PIP_2_ hydrolysis by PLCs in both *in cell* and *in vitro* assays. Their effect was critically dependent on charge valence of the cations, as predicted by the charge shielding model and as demonstrated by a shift of the dose-response curves of *in vitro* enzyme assays. Our results are consistent with a previous report: polyamines inhibited G protein- or Ca^2+^-mediated activation of phosphoinositide hydrolysis in GH3 cells [48]. In addition, Pina-Chable *et al* demonstrated that spermidine and spermine reduce the activity of PI-PLC from Madagascar periwinkle (*Catharanthus roseus)* above 10 μM [18]. It is worthwhile to mention that spermine (10–100 μM) stimulates the activity of a partially purified membrane PLC from Madagascar periwinkle, human recombinant PLCδ1 and rat liver PLCδ1 [14,16]. The stimulatory effect was postulated to be mediated by the region spanning highly conserved X and Y domains, but not PH-domain [49] and the putative binding region for spermine is rich in acidic and negatively charged amino acids such as glutamate or aspartate [50]. However, we did not observe any stimulatory effect of spermine at 10 and 100 μM on PLCβ1 activity at both 2 nM and 20 μM free [Ca^2+^] (Fig 5B and S5 Fig). In addition, the activity of PLCδ1 was not stimulated by 100 μM spermine either. However 100 μM spermine potentiated PLCγ1 activity only with a longer incubation time (> 12 min; S6 Fig). These conflicting results may be due to different assay conditions or sources for PLC and the issue needs to be clarified in the future studies.

Neomycin, an antibiotic, was proposed to bind to PIP_2_ with high affinity in isolated platelet membrane [51] and also inhibits thrombin-stimulated phosphoinositide turnover and initiation of cell proliferation [52]. The inhibitory effect of neomycin on PLC activity has been demonstrated in transformed roots of Madagascar periwinkle [18]. In our experiment, neomycin has the highest charge shielding effect on the activity of all PLCs tested here, apparently due to its large valence (+6).

Two phenomena in our study could not be explained by electrostatic charge shielding of PIP_2_. First, the inhibitory effect of neomycin but not spermine was partial. To explain it, we used W factor in our model, indicating incomplete shielding of PIP_2_ due to the large molecular size of neomycin and repulsion between these highly charged molecules. Secondly, although putrescine has the same valence as divalent cations, the inhibitory effect of putrescine (> 10 mM) is less than that of divalent cations (> 1 mM). In our model, we considered that putrescine has less than its original charge valence because the two charges are separated from each other ('charge separation effect'). Despite the adjustment, we could not fit the putrescine results well. We do not have a clear explanation at present. One possibility is that, being larger than divalent cations (ionic radius, < 0.1 nm), putrescine (< 1 nm) accumulates less efficiently around PIP_2_ (crowding effect). These two effects are considered as Y factor in our model.

### Physiological Significance of Charge Shielding Effect

**Fig 7:**
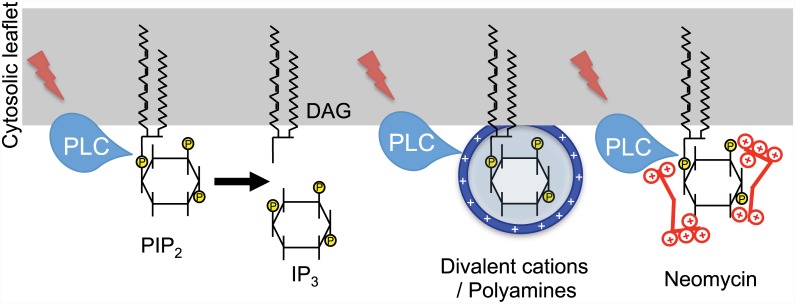
Working model for the modulation of PLCs activity via charge shielding of PIP_2_ by cations. (Left) After activation of PLC by G_q_-coupled GPCRs or receptor tyrosine kinases as indicated by lightning flash, PLC hydrolyzes PIP_2_ and generates intracellular second messengers, IP_3_ and DAG. (Middle) Positively charged divalent cations or polyamines accumulate around the negatively charged PIP_2_ and reduce electrostatic interaction between PIP_2_ and PLC, resulting less PIP_2_ hydrolysis. (Right) Highly charged neomycin inhibits PLC similarly. However the charge shielding by neomycin does not inhibit the PLCs activity completely, possibly due to their limited accumulation around PIP_2_ which, in turn, is caused by either too large size or too high charge density. See text for details.

Our data highlight the significance of the electrostatic charge shielding effect on the activity of PLCs (Fig 7). Modulation of PLCβ affects G_q_-coupled GPCR signaling, while modulation of PLCδ and PLCγ modifies diverse cellular functions, including receptor tyrosine kinase signaling, metastasis, cell migration, and brain disorders [53]. Does the charge shielding of PIP_2_ occur in physiological conditions? The answer is critically dependent on Mg^2+^ and polyamine concentration. Their intracellular levels are not well determined and may change dynamically. A significant portion of Mg^2+^ (total concentration ~ mM) is known to be bound to ATP, while polyamines (~100 μM) are coupled to negatively charged nucleic acids. Therefore even the additive charge shielding of PIP_2_ by divalent ions and polyamines may be marginal or just under critical levels. However, if their concentrations increase, enzyme activity of PLC would be lowered significantly and meaningfully by the cations under the specific conditions such as expression of TRPM7, G2 cell cycle phase or in cancer cells. In addition, our findings support the concept that electrostatic charge shielding phenomena are general mechanism and activity of many (if not all) other PIP_2_-interacting proteins would be controlled by the endogenous level of Mg^2+^ and polyamines as supported by previous studies [10,54,55]. Effect of polyamines on cellular signaling involving highly charged biomolecules such as IP_3_—IP_7_ (-4 - -8) needs to be investigated in light of charge shielding. Equally the shielding effect of negatively charged ions such as ATP (-4) on positively charged molecules or proteins would be an interesting subject of future studies.

## Supporting Information

S1 Fig Measurement of PLC activity with WH-15. (A) Real-time PLCγ1 and PLCδ1 activities were measured at 20 μM free Ca^2+^ concentration. *n* = 5 for each condition. Error bars are smaller than symbol sizes. (B) Activity of recombinant PLC proteins was estimated from the linear slope at 10 and 30 min for PLCγ1 and PLCδ1, respectively. The activity for PLCβ1 was measured in Fig 1C (30 min).
(EPS)

S2 Fig TRPM7 currents in HEK293-TRPM7 cells. Cells were incubated with or without 1 μg/mL tetracycline for 18–26 h to induce the expression of TRPM7 channels. The holding potential was 0 mV and the currents were recorded by applying a voltage-ramp from -100 mV to + 100 mV for 40 ms. To identify TRPM7-mediated current component, recordings were obtained from cells perfused with normal Ringer’s or extracellular 10 mM MgCl_2_ Ringer’s solution. In all measured cells, 10 mM MgCl_2_ Ringer's increased current by ~260–1,220 pA at -100 mV.
(EPS)

S3 Fig Effect of 1 mM extracellular MgCl_2_ on PLC activity in HEK293-TRPM7 cells. Cells were transfected with M_1_R and PH-GFP, and TRPM7 channels were activated by voltage clamp to accumulate divalent cations. The average rate of PIP_2_ hydrolysis by PLC was estimated by the monitoring of translocation of PH-GFP into the cytosol upon activation of M_1_R with 1 μM Oxo-M. For the accumulation of divalent cations into the cells, external solutions containing 1 mM MgCl_2_ (*N* = 4) was perfused at the indicated time, and their influx through TRPM7 was triggered by a negative membrane potential (-80 mV).
(EPS)

S4 Fig The effect of experimental manipulations on the interaction between M_1_R and Gα protein in HEK293-tsA201 cells. (Right Top) Schematic diagram of FRET analysis. Curved arrows symbolize the continuous recruitment of labeled trimeric G-proteins and their dissociation from an activated M_1_R. (A) Cell were transfected with M_1_R-CFP, YFP-G_αq_, G_β1_, and G_γ2_ and FRET signals were measured in unpatched cell (*N* = 7), patched cell without spermine (*N* = 5), and patched cells with 3 mM spermine (*N* = 5). (B) Summary of M_1_R-G_αq_ interaction with or without pipette perfusion of spermine. The results are mean ± SEM. N.S., not significant.
(EPS)

S5 Fig Spermine does not stimulate the activity of PLCβ1. Recombinant PLCβ1 protein (20 ng/reaction) was incubated with indicated concentrations of spermine with 20 nM or 20 μM free Ca^2+^ concentration for 90 min. *n* = 4 for each condition. ** *P* < 0.01 compared to control group (only PLCβ1 protein). N.S., not significant.
(EPS)

S6 Fig Spermine potentiates the activity of PLCγ1. Recombinant PLCγ1 protein (10 ng/reaction) was incubated with 100 μM of spermine with 20 μM free Ca^2+^ concentration for indicated incubation times. *n* = 2 for each condition. * *P* < 0.05 compared to control group (only PLCγ1 protein). N.S., not significant.
(EPS)

S1 Protocol Fluorescence resonance energy transfer (FRET) measurement. (PDF)

S1 Table Concentrations of free divalent cations in each conditions of PLC activity assay with WH-15. The base assay solution had 3 mM CaCl_2_ and 3 mM EGTA (16.4 μM free Ca^2+^) and its ionic strength is 0.073. The concentrations of free divalent cations were calculated by Maxchelator program (http://maxchelator.stanford.edu) under the conditions (20°C, pH 7.4, and 0.073 ionic strength).
(PDF)

## Abbreviations

DAG: diacylglycerol
IP_3_: inositol 1,4,5-triphosphate
M_1_R: M_1_ muscarinic receptor
Oxo-M: oxotremorine-M
PH: pleckstrin homology
PH-YFP: eYFP-PH-PLCδ1
PIP_2_: phosphatidylinositol 4,5-bisphosphate
PI-PLC: phosphoinositide-specific PLC
PLC: phospholipase C
