# Supplementary material for: Charge Shielding of PIP2 by Cations Regulates Enzyme Activity of Phospholipase C
Source: PLoS One. 2015 Dec 11;10(12):e0144432. doi: 10.1371/journal.pone.0144432 (PMC4676720; doi:10.1371/journal.pone.0144432)
Supplement: S1 Protocol — (PDF) [file pone.0144432.s007.pdf]

S1 Protocol. Fluorescence resonance energy transfer (FRET) measurement

Epifluorescence photometry was used to measure the FRET between CFP-M_1R and YFP-$G_{\alpha q}$. HEK293-tsA201 cells were transfected with the same amount of CFP-M_1R, $G_{\beta 1}$, and $G_{\gamma 2}$ together with YFP-$G_{\alpha q}$ (0.8 μg) using X-tremegene9 (Roche). After overexpression with all constructs together, we chose cells which have membrane localized CFP and YFP signal. We collected CFP or YFP emission *via* photomultipliers in photon-counting mode using an inverted Nikon diaphot microscope equipped with a 40X 1.3 NA oil-immersion objective [32]. CFP was excited at 440 nm. The bleed-through from CFP to the YFP channel was 17%, and the direct excitation of YFP by CFP excitation wavelength was negligible. FRET ratio (FRETr) was taken as the ratio of YFP emission divided by CFP emission after corrections. FRETr was normalized to remove cell-to-cell variation.
