# Supplementary material for: Charge Shielding of PIP2 by Cations Regulates Enzyme Activity of Phospholipase C
Source: PLoS One. 2015 Dec 11;10(12):e0144432. doi: 10.1371/journal.pone.0144432 (PMC4676720; doi:10.1371/journal.pone.0144432)
Supplement: S1 Table — The base assay solution had 3 mM CaCl2 and 3 mM EGTA (16.4 μM free Ca2+) and its ionic strength is 0.073. The concentrations of free divalent cations were calculated by Maxchelator program (http://maxchelator.stanford.edu) under the conditions (20°C, pH 7.4, and 0.073 ionic strength). (PDF) [file pone.0144432.s008.pdf]

S1 Table. Concentrations of free divalent cations in each conditions of PLC activity assay with WH-15.

Divalent cation (mM)		Free Ca^{2+} (µM)	Free Mg^{2+} (µM)	Free Ba^{2+} (µM)
$MgCl_2$	0	16.4	0.0	
	1	17.1	999.9	
	3	18.2	2,999.9	
	5	19.4	5,000.0	
	10	21.9	9,999.7	
	15	24.2	14,999.6	
$CaCl_2$	0.1	102.8		
	0.3	301.1		
	1	1,000.5		
	3	3,000.0		
	5	4,999.8		
	10	10,000.0		
	15	14,999.8		
$BaCl_2$	1	88.5		916.1
	3	152.5		2,851.7
	5	196.1		4,808.7
	15	333.2		14,673.2
